# Relationship between quantitative epicardial adipose tissue based on coronary computed tomography angiography and coronary slow flow

**DOI:** 10.1186/s12872-023-03541-z

**Published:** 2023-10-10

**Authors:** Jing Tong, Gui-Guang Bei, Li-Bo Zhang, Yu Sun, Miao Qi, Ben-Qiang Yang

**Affiliations:** Department of Radiology, General Hospital of Northern Theater Command, 83 Wenhua Road, Shenyang, 110016 Liaoning Province China

**Keywords:** Coronary slow flow, Epicardial adipose tissue, Coronary computed tomography angiography

## Abstract

**Background:**

The purpose of this study was to explore the relationship between quantitative epicardial adipose tissue (EAT) based on coronary computed tomography angiography (CCTA) and coronary slow flow (CSF).

**Methods:**

A total of 85 patients with < 40% coronary stenosis on diagnostic coronary angiography were included in this retrospective study between January 2020 and December 2021. A semi-automatic method was developed for EAT quantification on CCTA images. According to the thrombolysis in myocardial infarction flow grade, the patients were divided into CSF group (n = 39) and normal coronary flow group (n = 46). Multivariate logistic regression was used to explore the relationship between EAT and CSF. Receiver operating characteristic (ROC) curve was plotted to evaluate the diagnostic value of EAT in CSF.

**Results:**

EAT volume in the CSF group was significantly higher than that of the normal coronary flow group (128.83± 21.59 mL vs. 101.87± 18.56 mL, P < 0.001). There was no significant difference in epicardial fat attenuation index between the two groups (P > 0.05). Multivariate logistic regression analysis showed that EAT volume was independently related to CSF [odds ratio (OR) = 4.82, 95% confidence interval (CI): 3.06–7.27, P < 0.001]. The area under ROC curve for EAT volume in identifying CSF was 0.86 (95% CI: 0.77–0.95). The optimal cutoff value of 118.46 mL yielded a sensitivity of 0.80 and a specificity of 0.94.

**Conclusions:**

Increased EAT volume based on CCTA is strongly associated with CSF. This preliminary finding paves the way for future and larger studies aimed to definitively recognize the diagnostic value of EAT in CSF.

**Supplementary Information:**

The online version contains supplementary material available at 10.1186/s12872-023-03541-z.

## Background

Contrast agent flow in coronary angiography acts like coronary blood flow, and the distal vessel opacification may be delayed despite the absence of obstructive coronary artery disease. This phenomenon of coronary angiography is commonly referred to as primary coronary slow flow (CSF) phenomenon. It has been considered a specific disease entity, however, whether it should be considered a specific coronary syndrome or a stage in the development of coronary microvascular dysfunction remains undefined [1, 2]. CSF was first proposed by Tambe et al. [3] in 1972, and its diagnosis was based on the results of coronary angiography. At present, the most applicable diagnostic standard was proposed by Beltrame [4]. CSF is characterized by normal or near normal epicardial coronary arteries (stenosis < 40%) with delayed distal vessel contrast opacification as evidenced by either thrombolysis in myocardial infarction (TIMI) 2 flow or corrected TIMI frame count > 27 frames (30 frames/s) in at least one epicardial vessel. The incidence of CSF is about 1-7% in patients with suspected coronary heart disease undergoing coronary angiography [5]. Angina pectoris is the most common clinical presentation of CSF patients, which affects their quality of life and even leads to ventricular tachyarrhythmias or cardiac death [6–8]. The aetiology and pathogenesis of CSF are not clearly defined, and there is no effective non-invasive examination.

Epicardial adipose tissue (EAT) is a visceral fat deposit between the myocardium and the visceral pericardium. EAT has paracrine effects. Released cytokines such as interleukin (IL)-6, IL-1β and tumour necrosis factor (TNF)-α diffusing and accumulating in the epicardial vascular wall may induce local inflammatory reactions that potentially result in endothelial dysfunction [9]. The latter leads to the increase of coronary vascular resistance and thus reduces coronary blood flow [10, 11]. Fat attenuation index (FAI) is an AI-driven quantitation of adipose tissue radiodensity which is computationally adjusted for a range of additional factors, such as CT technical parameters and adipocyte morphology [12, 13]. Coronary computed tomography angiography (CCTA) is a commonly used non-invasive imaging modality to identify and measure EAT. Presently, no research evaluating EAT of CSF patients based on CCTA has been reported. In this study, we used CCTA for measuring the EAT volume (EATV) and FAI to investigate the relationship between EAT and CSF.

## Methods

### Study population

**Fig. 1:**
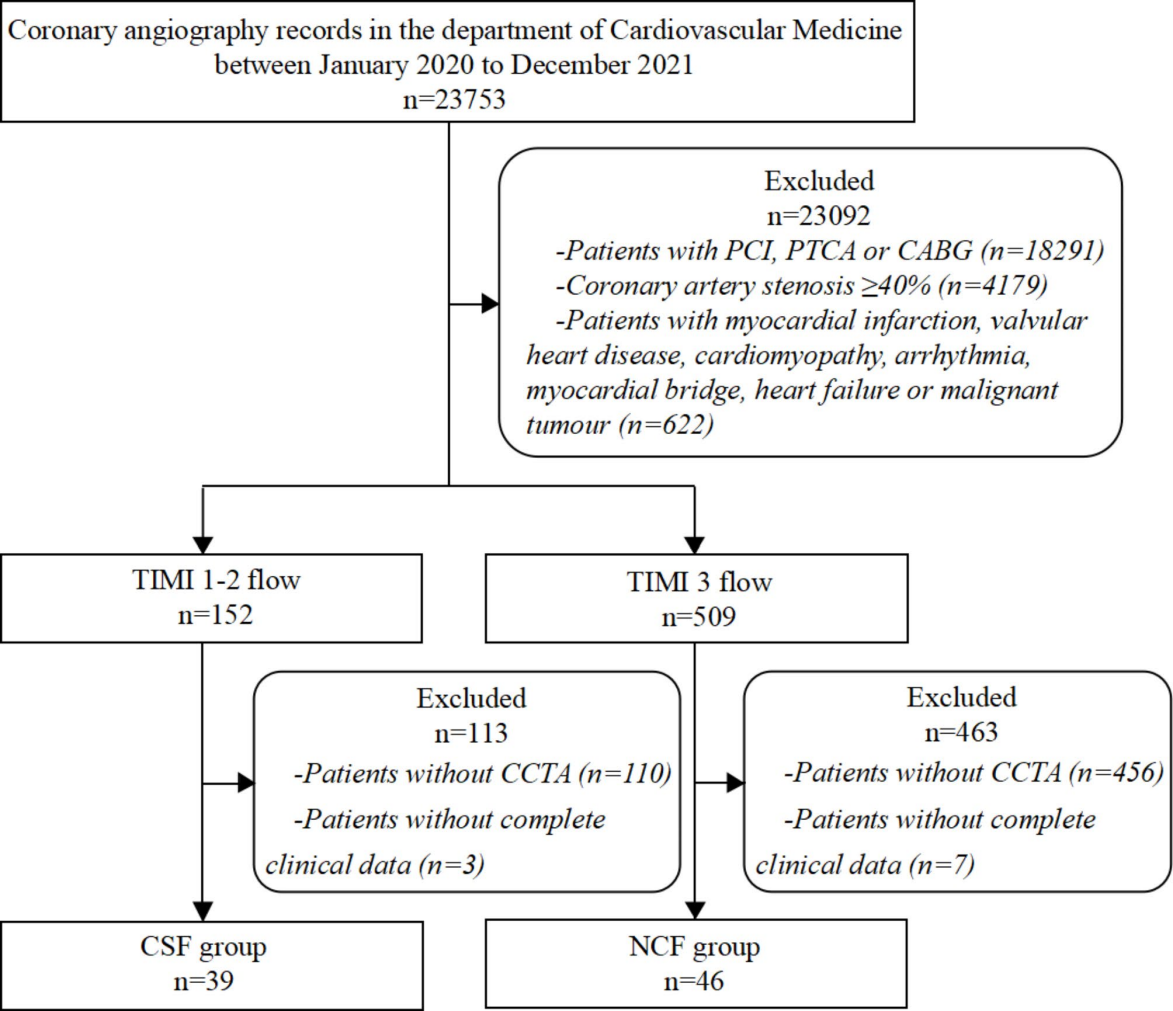
Flow diagram of the participants. *PCI* percutaneous coronary intervention, *PTCA* percutaneous transluminal coronary angioplasty, *CABG* coronary artery bypass grafting, *TIMI* thrombolysis in myocardial infarction, *CCTA* coronary computed tomography angiography, *CSF* coronary slow flow, *NCF* normal coronary flow

All coronary angiography records (n = 23,753) at the Department of Cardiology in General Hospital of Northern Theater Command between January 2020 and December 2021 were retrospectively collected. All patients underwent diagnostic coronary angiography for suspected coronary artery disease. Patients with percutaneous coronary intervention (n = 16,648), percutaneous transluminal coronary angioplasty (n = 1,584) or coronary artery bypass grafting (n = 59), coronary artery stenosis ≥ 40% (n = 4,179), myocardial infarction, heart valve disease, cardiomyopathy, arrhythmia, myocardial bridge, heart failure and malignant tumor (n = 622) were excluded. According to TIMI coronary flow grade, all patients were divided into two groups: CSF group (n = 152) with TIMI 1–2 flow and normal coronary flow group (NCF group, n = 509) with TIMI 3 flow. Both groups excluded patients who had not undergone CCTA or CCTA time distance coronary angiography more than 1 month (n = 110 in CSF group, n = 456 in NCF group) and had incomplete clinical information (n = 3 in CSF group, n = 7 in NCF group). We finally enrolled 39 subjects in CSF group and 46 subjects in NCF group (Fig. 1).

### Clinical data

General data of patients were collected, including age, sex, body mass index (BMI), previous history and laboratory test results within 3 days before coronary angiography. In addition, left ventricular (LV) function assessed by transthoracic echocardiography within 24 h before coronary angiography was obtained from hospital records, including LV end-diastolic diameter, LV ejection fraction (LVEF), and the ratio of diastolic mitral inflow velocity E peak and A peak (E/A).

### CCTA data acquisition

CCTA was performed on a 256-slice CT scanner (Brilliance iCT, Philips Healthcare, Cleveland, OH, USA). Prospective ECG-gated scan was performed and all images were reconstructed using iterative reconstruction method. About 60–70 mL of iodine contrast agent was intravenously injected through the cubital vein at a rate of 4.5–5.5 mL/s using a high-pressure injector, followed by 20–30 mL of saline flushing at the same flow rate. A bolus tracking technique was used with a trigger threshold of 100 Hounsfeld units (HU) at the descending aorta. The scan parameters were set as follows: tube voltage 120 kV, tube current modulation technique, detector collimation 128 × 0.625 mm, pitch 0.16, slice thickness 0.9 mm, section increment 0.45 mm, rotation time 0.27 s, field of view 180 × 180 mm, matrix 512 × 512.

### EATV and epicardial FAI measurement

EAT was quantitatively measured on axial CCTA images at 75% of the R-R interval. Pericardium was semi-automatically segmented and EAT was measured with a software tool as reported previously [14] from our group. Without knowledge of the trial design and clinical data, a radiologist with 5 years’ experience in coronary imaging diagnosis automatically segmented the pericardial contour with software and manually small scale enlarged or reduced the image annotation range to ensure a good match with the anatomical structure. Then a radiologist with more than 10 years’ experience in coronary imaging diagnosis checked and modified the segmentation result, and EAT parameters were extracted. The upper boundary of the pericardium was the bifurcation of pulmonary trunk, and the lower boundary was the apex cordis.

**Fig. 2:**
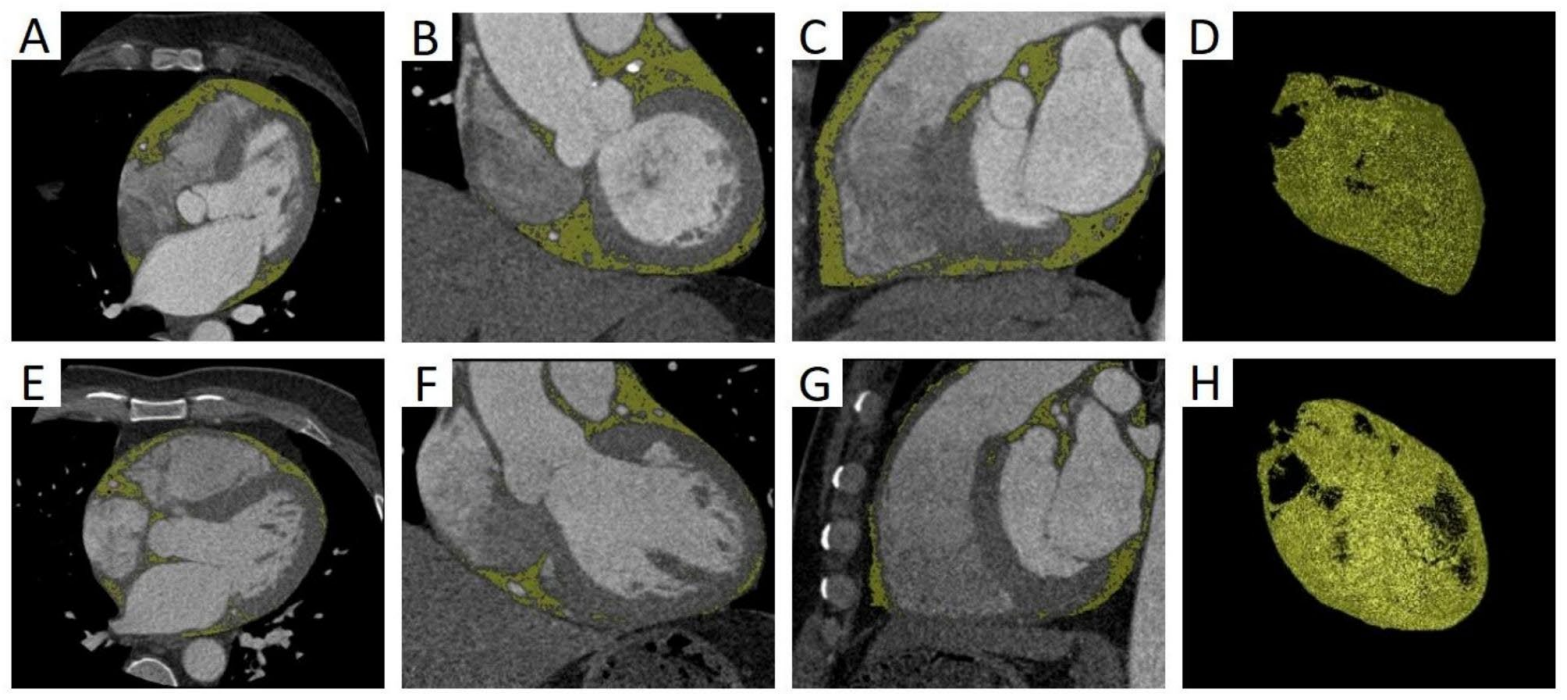
Semi-automatic quantitative measurement of EAT on CCTA images. (A-D) EAT on axial, coronal, sagittal and three-dimensional reconstruction images in CSF group. EATV was 155.45 mL. (E-H) EAT on axial, coronal, sagittal and three-dimensional reconstruction images in NCF group. EATV was 104.29 mL

EAT was defined as all voxels with attenuation between − 190 HU and − 30 HU, and epicardial FAI was defined as the average CT attenuation of the adipose tissue within the pre-specified attenuation window of -190 HU to -30 HU. Our software was used to identify EAT and quantify its parameters (Fig. 2).

### Statistical analysis

Continuous variables were presented as mean ± standard deviation (SD) or median [interquartile range (IQR)]. Categorical variables were presented as absolute values and percentages. Continuous variables were compared by independent t-test or Mann-Whitney U test, and categorical variables were compared by chi-square test. The variables with significant differences in the univariate analysis were included in the multivariate logistic regression to determine the independent factors. Receiver operating characteristic (ROC) curve was plotted, and the area under the ROC curve (AUC) was calculated to evaluate the diagnostic value. The optimal cutoff value was determined by the maximum Youden index. Statistical analysis was performed with SPSS Statistics v.26 software (IBM, Chicago, IL, USA). Two-sided tests were used and *P* < 0.05 was considered to be statistically significant.

## Results

### Basic clinical data

There were no statistically significant differences in age, gender, BMI, history of hypertension and smoking between CSF group and NCF group (*P* > 0.05, Table 1).

**Table 1:** Clinical data comparison of patients in CSF and NCF groups

Variables	CSF group (n = 39)	NCF group (n = 46)	t/χ^2^/Z	*P*-value
Cardiovascular risk factors				
Age (years)	59.82 ± 9.67	57.54 ± 10.85	−1.01^a^	0.314
Male, n (%)	28 (71.79)	29 (63.04)	0.73^b^	0.392
BMI (kg/m^2^)	25.52 ± 3.49	24.65 ± 3.58	−1.13^a^	0.261
WBC (×10^9^/L)	5.80 [5.20–7.70]	6.50 [5.65–8.25]	−1.77^c^	0.078
NEU (×10^9^/L)	3.59 [3.07–4.70]	3.93 [3.19–5.56]	−1.77^c^	0.077
HCY (umol/L)	10.88 [10.07–13.46]	9.43 [8.06–11.84]	−2.74^c^	0.006
CHOL (mmol/L)	4.36 ± 1.14	3.78 ± 0.90	−2.66^a^	0.009
HDL-C (mmol/L)	1.20 ± 0.30	1.14 ± 0.25	−0.93^a^	0.354
LDL-C (mmol/L)	2.39 ± 0.77	2.03 ± 0.71	−2.26^a^	0.026
Hypertension, n (%)	23 (58.97)	21 (45.65)	1.50^b^	0.221
Smoking, n (%)	24 (61.54)	20 (43.48)	2.76^b^	0.097
CCTA				
EATV (mL)	128.83 ± 21.59	101.87 ± 18.56	−6.83^a^	< 0.001
Epicardial FAI (HU)	−84.79 ± 5.96	−83.48 ± 6.53	0.96^a^	0.342
Echocardiography				
LV diameter (mm)	49.04 ± 4.53	47.47 ± 3.14	−1.60^a^	0.115
LVEF	0.64 ± 0.06	0.63 ± 0.04	2.68^a^	0.269
E/A	0.84 ± 0.29	0.92 ± 0.33	0.95^a^	0.345

The data are expressed as n (%) or mean± SD or median [Q1-Q3]

*CSF* coronary slow flow, *NCF* normal coronary flow, *BMI* body mass index, *WBC* white blood cells, *NEU* neutrophils, *HCY* homocysteine, *CHOL* serum total cholesterol, *HDL-C* high-density lipoprotein cholesterol, *LDL-C* low-density lipoprotein cholesterol, *CCTA* coronary computed tomography angiography, *EATV* epicardial adipose tissue volume, *FAI* fat attenuation index, *LV* left ventricular, *LVEF* left ventricular ejection fraction, *E/A* the ratio of diastolic mitral inflow velocity E peak and A peak

^a^ indicates t-value; ^b^ indicates χ^2^-value; ^c^ indicates Z-value

### Coronary angiographic characteristics

In 20 of 39 CSF patients, coronary angiography revealed three vessels including left anterior descending (LAD), left circumflex (LCx), and right coronary artery (RCA) with delayed distal vessel contrast opacification. TIMI 2 was present in 12 of the 20 patients, and TIMI 1–2 was present in 8 patients. In 19 of 39 CSF patients, coronary angiography revealed single vessel with delayed distal vessel contrast opacification, including LAD affected in 15 patients (TIMI 2 in 13 patients, TIMI 1–2 in 2 patients), LCx affected in 1 patient (TIMI 2), and RCA affected in 3 patients (TIMI 2 in all patients).

### EAT, echocardiographic, and laboratory parameters

EATV in CSF group was greater than that in NCF group with statistical significance (*Z* = -6.83, *P* < 0.001), whereas there was no significant difference in epicardial FAI between the two groups (*P* > 0.05) (Table 1). The levels of homocysteine, serum total cholesterol and low-density lipoprotein in CSF group were higher than those in NCF group with statistical significance (t/Z = -2.74, -2.66, -2.26, *P* = 0.006, 0.009, 0.026), whereas other laboratory indicators and echocardiographic parameters showed no significant differences (all *P* > 0.05) (Table 1).

### The association between EATV and CSF

Univariate and multivariate logistic regression analysis showed that EATV was independently related to CSF (*OR* 4.82, 95% CI 3.06–7.27, *P* < 0.001) (Table 2).

**Table 2:** Univariate and multivariate logistic regression analysis of factors for CSF

Variables	Univariable analysis		Multivariable analysis (univariable P < 0.05)	
	OR (95% CI)	*P*-value	OR (95% CI)	*P*-value
Age	0.97 (0.90; 1.06)	0.512		
Gender	1.02 (0.91; 1.44)	0.093		
BMI	0.96 (0.77; 1.21)	0.235		
WBC	1.47 (0.83; 2.02)	0.539		
NEU	0.54 (0.24; 1.99)	0.354		
HCY	1.13 (1.01; 1.26)	0.006	1.10 (0.97; 1.24)	0.154
CHOL	1.95 (1.00; 3.62)	0.014	1.93 (0.92; 3.57)	0.206
HDL-C	1.13 (0.93; 2.25)	0.302		
LDL-C	0.76 (0.28; 0.97)	0.049	0.80 (0.33; 1.96)	0.810
Hypertension	1.08 (0.79; 1.85)	0.931		
Smoking	1.70 (0.96; 2.83)	0.63		
EATV	4.80 (3.07; 7.24)	< 0.001	4.82 (3.06; 7.27)	< 0.001
Epicardial FAI	1.07 (0.93; 1.23)	0.319		
LV diameter	1.08 (0.84; 1.38)	0.149		
LVEF	0.83 (0.67; 1.20)	0.175		
E/A	1.22 (0.68; 2.29)	0.383		

*BMI* body mass index, *WBC* white blood cells, *NEU* neutrophils, *HCY* homocysteine, *CHOL* serum total cholesterol, *HDL-C* high-density lipoprotein cholesterol, *LDL-C* low-density lipoprotein cholesterol, *EATV* epicardial adipose tissue volume, *FAI* fat attenuation index, *LV* left ventricular, *LVEF* left ventricular ejection fraction, *E/A* the ratio of diastolic mitral inflow velocity E peak and A peak, *OR* odds ratio, *CI* confidence interval

### Diagnostic performance of EATV for identifying CSF

**Fig. 3:**
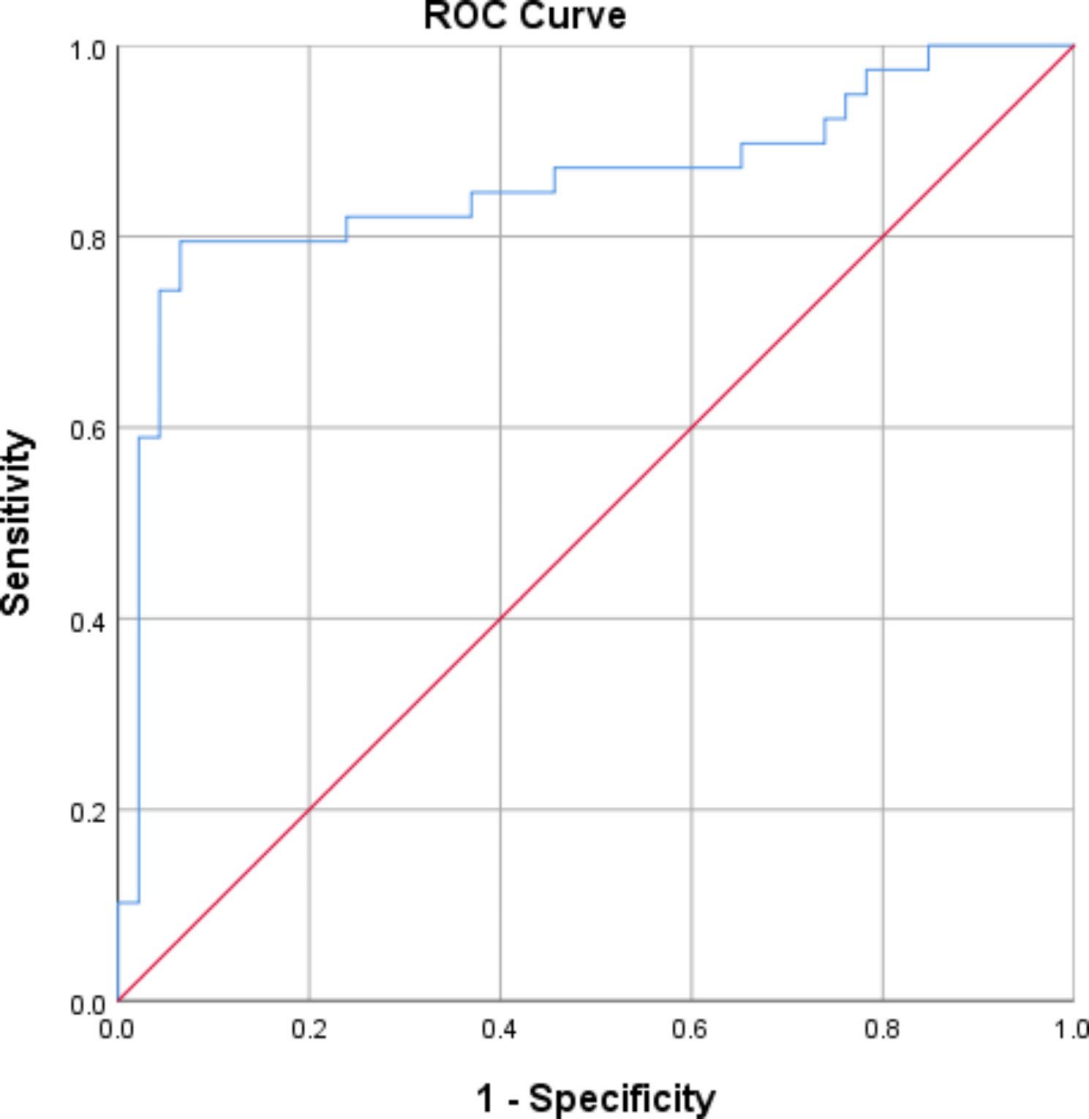
The receiver operating characteristic (ROC) curve of EATV for identifying CSF

The ROC curve of EATV for identifying CSF was shown in Fig. 3. The AUC for EATV in identifying CSF was 0.86 (95% CI: 0.77–0.95), indicating a moderate diagnostic performance. The optimal cutoff value of 118.46 mL yielded a sensitivity of 0.80 (95% CI: 0.72–0.92) and a specificity of 0.94 (95% CI: 0.77–0.97).

## Discussion

This study revealed the relationship between EAT and CSF. EATV in CSF group was greater than that in NCF group with statistical significance and multivariate logistic regression analysis showed that EATV was independently related to CSF. To the best of our knowledge, this is the first study to explore the relationship between EAT based on CCTA and CSF. There are several previous studies on the relationship between EAT thickness based on two-dimensional echocardiography and CSF [15–18]. Erdogan et al. [15] and Comert et al. [16] analyzed 20 and 33 CSF patients respectively, and found that echocardiographic EAT thickness was positively associated with CSF. Wu et al. [17] analyzed 16 CSF patients and found that echocardiographic EAT thickness was an independent predictor for CSF and was negatively associated with coronary TIMI flow grade. Weferling et al. [18] analyzed 48 CSF patients over a 10-year time period and found that echocardiographic EAT thickness in CSF patients was significantly higher than that in patients with normal coronary flow, but this appeared to have no effect on the long-term prognosis. Two-dimensional echocardiography provided a single linear measurement, but was unable to provide EATV data. Nerlekar et al. [19] found that the intra- and inter-observer repeatabilities were better for CT measurement of EAT than for echocardiography measurement. FAI is an index associated with vascular inflammation [13]. We found that there was no significant difference in epicardial FAI between CSF group and NCF group. This was possibly because the paracrine inflammatory signals from the epicardial vascular walls in CSF were not sufficient to cause a significant increase in the density of EAT. In this study, the LAD was the most commonly involved vessel in CSF patients, which was consistent with the most of previous studies [20, 21]. However, Hawkins et al. [22] found that the LAD was not the main vessel involved in CSF patients, and the involvement of three vessels was evenly distributed. They explained this finding with the higher burden of cardiovascular risk in their population, which might lead to a more diffuse vessel involvement.

Several studies have found that EAT was correlated with the occurrence and severity of coronary artery disease [23, 24]. It remains controversial, however, whether CSF could be considered as the primary stage of coronary artery disease [22, 25]. At present, the pathogenesis of CSF is not clearly defined. Multiple studies have shown high levels of inflammatory cytokines such as IL-1β and TNF-α in CSF patients and a strong positive correlation with coronary TIMI flow grade [26–28], indicating that inflammatory mechanisms may be involved in the development of CSF. EAT secretes a large amount of bioactive molecules that affect energy metabolism and vascular inflammation, including inflammatory cytokines such as IL-6, IL-1β and TNF-α [9]. In physiological state, EAT mediates positive effects, keeps the balance of anti- and pro-inflammatory, and maintains normal structure and function of the myocardium and coronary arteries. While in pathological state, EAT releases a large amount of inflammatory cytokines, shifts the balance toward pro-inflammatory state, and interacts with myocardium and coronary arteries via paracrine and vasocrine pathways, thereby interfering with the metabolism and function of coronary arteries [29, 30]. Whether inflammatory cytokines released by EAT lead to CSF via certain pathological mechanisms requires further confirmation.

In addition, this study found that the level of homocysteine in CSF group was higher than that in NCF group with statistical significance. Homocysteine is a sulfur-containing amino acid. Studies showed that elevated homocysteine level can lead to vascular endothelial injury, resulting in CSF [31, 32]. Demirci et al. [33] found that the level of homocysteine was significantly elevated in CSF group. They suggested that hyperhomocysteinemia may induce CSF endothelial dysfunction via direct toxic effects on endothelial cells, and may also indirectly induce endothelial dysfunction via inhibiting the activity of dimethylarginine dimethylaminohydrolase and reducing the synthesis of nitric oxide. Moreover, hyperhomocysteinemia may damage endothelial cells via oxidative stress induction, thereby causing CSF [34]. In this study, the levels of serum total cholesterol and low-density lipoprotein in CSF group were higher than those in NCF group with statistical significance, which were consistent with the previous research results [35, 36].

There were certain limitations in this study. First, this was a single-center retrospective study with a relatively small sample size. The single-center nature of the study limited the ability to generalize findings to a broader population. The retrospective nature of the study limited the establishment of causality. Since the study was cross-sectional, it couldn’t establish a temporal relationship between EAT and the onset of CSF. Secondly, the medication of patients was not taken into account. Statins have been reported to be associated with a reduction in epicardial fat accumulation, especially when given in high doses [37]. However, in this study, CSF patients were given moderate doses. Finally, this study did not provide follow-up data. Further studies with larger sample sizes are needed to improve statistical power. We will further assess how EAT could be used for early diagnosis, mechanism research, treatment and prognosis in CSF patients.

## Conclusions

EATV based on CCTA is strongly associated with CSF, and further studies are necessary to determine the role of EAT in the genesis and development of CSF.

### Electronic supplementary material

Below is the link to the electronic supplementary material.

Supplementary Material 1

## Data Availability

The datasets used and/or analysed during the current study are available from the corresponding author on reasonable request.

## Abbreviations

CSF: Coronary slow flow
TIMI: Thrombolysis in myocardial infarction
EAT: Epicardial adipose tissue
IL: Interleukin
TNF: Tumour necrosis factor
CCTA: Coronary computed tomography angiography
EATV: Epicardial adipose tissue volume
FAI: Fat attenuation index
NCF: Normal coronary flow
BMI: Body mass index
LV: Left ventricular
LVEF: Left ventricular ejection fraction
E/A: The ratio of diastolic mitral inflow velocity E peak and A peak
ROC: Receiver operating characteristic
AUC: Area under the ROC curve
LAD: Left anterior descending
LCx: Left circumflex
RCA: Right coronary artery
