# Supplementary material for: Relationship between quantitative epicardial adipose tissue based on coronary computed tomography angiography and coronary slow flow
Source: BMC Cardiovasc Disord. 2023 Oct 10;23:500. doi: 10.1186/s12872-023-03541-z (PMC10566056; doi:10.1186/s12872-023-03541-z)
Supplement: Supplementary file 1 — Supplementary Material 1 [file 12872_2023_3541_MOESM1_ESM.doc]

STROBE Statement—Checklist of items that should be included in reports of ***case-control studies***

|  | Item No | Recommendation |
| --- | --- | --- |
| **Title and abstract** | 1 | 1. Indicate the study’s design with a commonly used term in the title or the abstract Pages 1-2 |
|  |  | (*b*) Provide in the abstract an informative and balanced summary of what was done and what was found Pages 1-2 |
| Introduction | | |
| Background/rationale | 2 | Explain the scientific background and rationale for the investigation being reported Pages 2-4 |
| Objectives | 3 | State specific objectives, including any prespecified hypotheses Page 4, Lines71-72 |
| Methods | | |
| Study design | 4 | Present key elements of study design early in the paper Page 4 |
| Setting | 5 | Describe the setting, locations, and relevant dates, including periods of recruitment, exposure, follow-up, and data collection Pages 4-5 |
| Participants | 6 | (*a*) Give the eligibility criteria, and the sources and methods of case ascertainment and control selection. Give the rationale for the choice of cases and controls Page 4 |
|  |  | (*b*) For matched studies, give matching criteria and the number of controls per case Pages 4-5 |
| Variables | 7 | Clearly define all outcomes, exposures, predictors, potential confounders, and effect modifiers. Give diagnostic criteria, if applicable Pages 5-7 |
| Data sources/ measurement | 8* | For each variable of interest, give sources of data and details of methods of assessment (measurement). Describe comparability of assessment methods if there is more than one group Pages 5-7 |
| Bias | 9 | Describe any efforts to address potential sources of bias Pages 5-7 |
| Study size | 10 | Explain how the study size was arrived at Pages 4-5 |
| Quantitative variables | 11 | Explain how quantitative variables were handled in the analyses. If applicable, describe which groupings were chosen and why Pages 4-7 |
| Statistical methods | 12 | (*a*) Describe all statistical methods, including those used to control for confounding Pages 7-8 |
|  |  | (*b*) Describe any methods used to examine subgroups and interactions Pages 7-8 |
|  |  | (*c*) Explain how missing data were addressed Pages 7-8 |
|  |  | (*d*) If applicable, explain how matching of cases and controls was addressed Pages 7-8 |
|  |  | (*e*) Describe any sensitivity analyses Pages 7-8 |
| Results | | |
| Participants | 13* | (a) Report numbers of individuals at each stage of study—eg numbers potentially eligible, examined for eligibility, confirmed eligible, included in the study, completing follow-up, and analysed Pages 4-5 |
|  |  | (b) Give reasons for non-participation at each stage Pages 4-5 |
|  |  | (c) Consider use of a flow diagram Page 5 |
| Descriptive data | 14* | (a) Give characteristics of study participants (eg demographic, clinical, social) and information on exposures and potential confounders Pages 8-10 |
|  |  | (b) Indicate number of participants with missing data for each variable of interest Pages 8-10 |
| Outcome data | 15* | Report numbers in each exposure category, or summary measures of exposure Pages 10-11 |
| Main results | 16 | (*a*) Give unadjusted estimates and, if applicable, confounder-adjusted estimates and their precision (eg, 95% confidence interval). Make clear which confounders were adjusted for and why they were included Page 11 |
|  |  | (*b*) Report category boundaries when continuous variables were categorized Pages 8-11 |
|  |  | (*c*) If relevant, consider translating estimates of relative risk into absolute risk for a meaningful time period Pages 10-11 |

| Other analyses | 17 | Report other analyses done—eg analyses of subgroups and interactions, and sensitivity analyses  Page 12 |
| --- | --- | --- |
| Discussion | | |
| Key results | 18 | Summarise key results with reference to study objectives Pages 13-15 |
| Limitations | 19 | Discuss limitations of the study, taking into account sources of potential bias or imprecision. Discuss both direction and magnitude of any potential bias Page 15 |
| Interpretation | 20 | Give a cautious overall interpretation of results considering objectives, limitations, multiplicity of analyses, results from similar studies, and other relevant evidence Pages 13-15 |
| Generalisability | 21 | Discuss the generalisability (external validity) of the study results Page 15 |
| Other information | | |
| Funding | 22 | Give the source of funding and the role of the funders for the present study and, if applicable, for the original study on which the present article is based Page 17 |

*Give information separately for cases and controls.

**Note:** An Explanation and Elaboration article discusses each checklist item and gives methodological background and published examples of transparent reporting. The STROBE checklist is best used in conjunction with this article (freely available on the Web sites of PLoS Medicine at http://www.plosmedicine.org/, Annals of Internal Medicine at http://www.annals.org/, and Epidemiology at http://www.epidem.com/). Information on the STROBE Initiative is available at http://www.strobe-statement.org.
